# Tracking cats revisited: Placing terrestrial mammalian carnivores on *δ*^2^H and *δ*^18^O isoscapes

**DOI:** 10.1371/journal.pone.0221876

**Published:** 2019-09-03

**Authors:** Geoff Koehler, Keith A. Hobson

**Affiliations:** 1 NHRC Stable Isotope Laboratory, Environment and Climate Change Canada, Saskatoon, SK, Canada; 2 Department of Biology, University of Western Ontario, London, ON, Canada; University of Oregon, UNITED STATES

## Abstract

The relationship between hydrogen and oxygen stable isotopic compositions in environmental water and hair was investigated for both domestic cats (*Felis catus*) and dogs (*Canis lupus familiaris*). A strong, but different, covariance was measured between the hydrogen and oxygen stable isotopic compositions of local precipitation and hair keratin from both cats and dogs. These isotopic differences are most likely a result of the differences between the dietary and drinking water needs of cats compared to dogs. Moreover, the *δ*^2^H and *δ*^18^O values of hair from captive and wild felids and canids, such as cougars (*Puma concolor*), bobcats (*Lynx rufus*), and wolves (*Canis lupus*) are broadly consistent with these measurements. This evidence indicates that while the water budgets of terrestrial mammalian carnivores need to be considered in isotopic applications, it is clear that felids and canids may be placed on tissue–specific hydrogen and oxygen isotopic landscapes for ecological, provenance, or forensic studies.

## Introduction

Global populations of terrestrial carnivores are increasingly threatened by habitat loss, reduction of prey, and direct hunting by humans for food, traditional curatives, predator control, and trophies [1, 2]. Of these, many species belonging to Felidae, Canidae, and Ursidae have suffered the largest and most severe population declines and range contraction [3, 4]. The numbers are shocking, with recent dramatic population and range declines being reported for most wild mammalian carnivores, including such charismatic and iconic species as lions (*Panthera leo*) [5, 6] and cheetahs (*Acinonyx jubatus*) [7]. Despite ongoing restorative conservation efforts, red wolves (*Canis rufus*) were declared extinct in the wild in 1980 [8] and tigers (*Panthera tigris*) are predicted to be functionally extinct within a few decades [9, 10]. Because of their large range, some carnivores, such as cougars (*Puma concolor*) and brown bears (*Ursus actos*), are listed as species of least concern on the IUCN (International Union for the Conservation of Nature) Red List. However, it is evident that even these species have seen range contraction and population declines in recent decades [11].

As a consequence, many of these species, particularly the large African felids, are the focus of wildlife sanctuaries and thus protected from human activities such as hunting and habitat destruction. Despite these conservation endeavours, such species continue to be imperiled by human activities, many of which revolve around trophy hunting and illegal trade in wildlife and their parts. To combat this, forensic and ecological tools are required to trace tissues to potential geographic origins. In this respect, the hydrogen and oxygen stable isotopic compositions of animal tissues hold great potential for conservation in the form of forensic markers [12], provenance estimation [13], or determination of migration patterns of wildlife [14]. This is because the H and O stable isotopic compositions of metabolically inert animal tissues, such as hair, hooves, feathers, and horns, are related to those of body water, and thus ingested environmental waters [15]. Because the H and O stable isotopic compositions of water varies regionally, so do those of animal tissues. This is the main premise which links organisms with their environment through isotopic landscapes, or isoscapes [16], and has been used in many ecological studies [17, 18].

However, while a relationship exists between the H and O stable isotopic compositions of animal tissues and local water, it is complicated by the metabolic and physiological-chemical processes involved in the transfer of hydrogen and oxygen from environmental water to animal tissues [19]. This becomes particularly acute for carnivores who acquire a substantial portion of their body water from food, rather than from drinking water. This was the conclusion of Pietsch *et al*. [20] who reported that because of these physiological effects, the hydrogen and oxygen stable isotopic compositions of cougar and bobcat (*Lynx rufus*) hair in museum specimens had little relationship to those of environmental water and so are not valuable for forensic tracking. However, because their conclusion conflicts with other studies [21, 22] and because the use of isotopic methods to elucidate the provenance of carnivores is theoretically strong, we felt that a re-evaluation of the isotopic coupling between mammalian carnivores and environmental water was necessary but using modern unpreserved samples rather than museum specimens.

Ideally, to test this would require an inventory of hair or claw samples from a large number of wild mammalian carnivores from locations that have disparate stable isotopic compositions of environmental water. Obviously, this would be a large and expensive undertaking as wild carnivores tend to be found in remote locales and are, for the most part, reclusive and possibly dangerous. As a proxy, we collected hair from two common domesticated carnivores: cats (*Felis catus*) and dogs (*Canis lupus familiaris*) from urban animal shelters in different locations across North America. We assume that, isotopically speaking, domestic cats and dogs are physiologically close enough to their wild cousins that the processes responsible for isotopic coupling between hair and environmental water would be similar and would allow us to evaluate the application of these isotopic methods to wild mammalian carnivores.

## Experimental

### Samples

**Fig 1:**
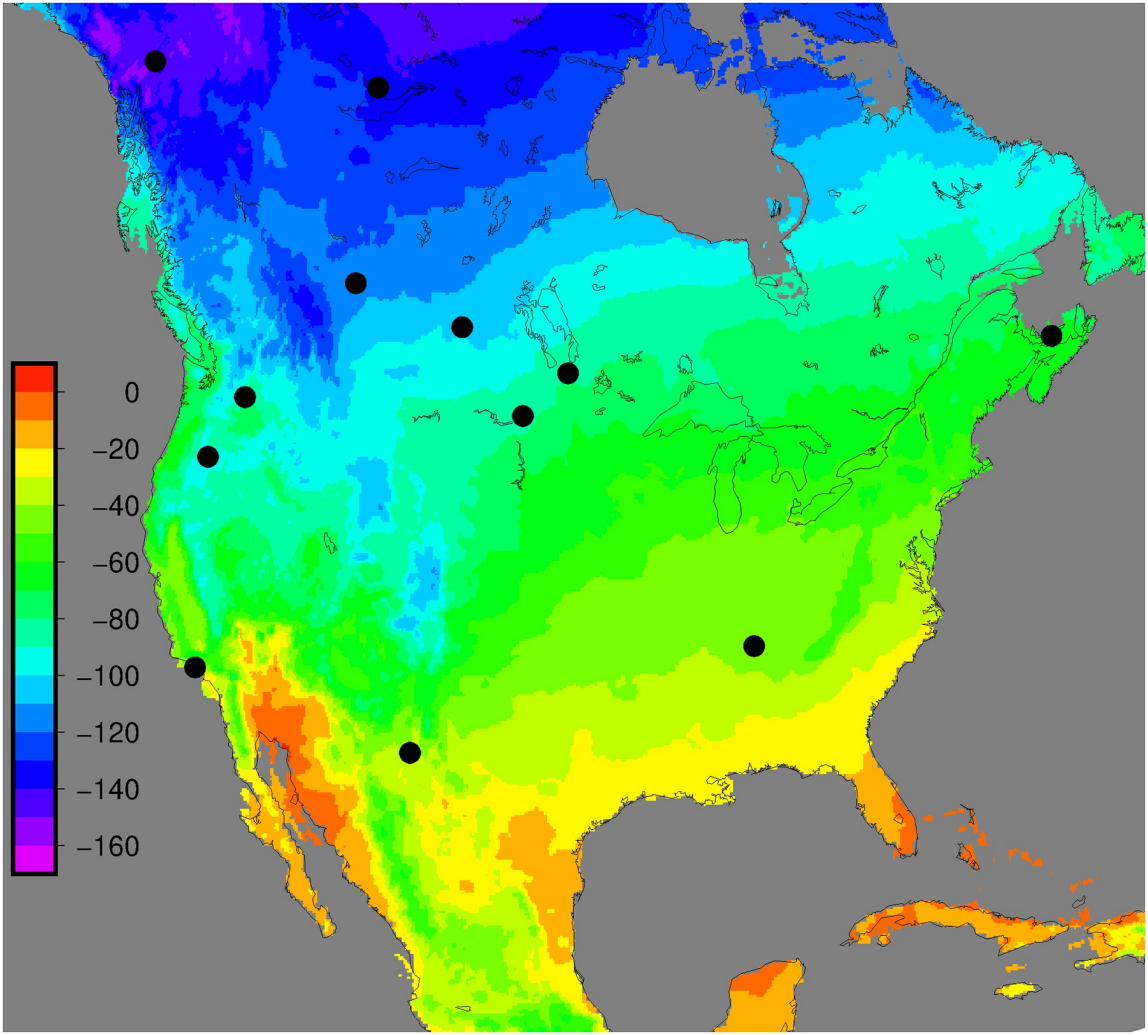
Locations of participating shelters. Coloured contours represent the hydrogen isotopic composition of growing-season precipitation from the RCWIP model program, IAEA [23].

Adult cat (n = 112) and dog (n = 124) guard hair samples were collected into paper envelopes by participating animal shelters (Fig 1) and forwarded to the NHRC lab in Saskatoon. Shelters were asked to provide about 0.5g of hair and to sample approximately equal numbers of cats and dogs. Meta-data collected from each animal were: ID, species, gender, approximate age, food (wet or dry), approximate length of time the animal was resident in the shelter, and general health or body condition. A sample of local water used in the shelter was also collected into supplied 2 mL bottles.

For comparison to these domestic animals, hair samples were also collected from both wild and captive cougars (n = 8), bobcats (n = 10), tigers (n = 1), wolves (*Canis lupus*, n = 2), coyotes (*Canis latrans*, n = 1), foxes (*Vulpes vulpes*, n = 2), and lions (n = 1) from participating zoos and also from existing collections within our laboratory. For zoos, hair was collected from the animal enclosure or directly from the animal during veterinary procedures. Untanned cougar hair and/or claw samples were also obtained from participating museums, where available.

### Stable isotope analysis

Stable isotopic compositions of all hair and water samples were measured at the NHRC Stable Isotope Laboratory of Environment and Climate Change Canada in Saskatoon, Canada. Prior to analyses, hair samples were cleaned of debris and any surface oils were removed by rinsing in 2:1 chloroform:methanol and finally homogenized to powder with a ball grinder (Retsch model MM-301, Haan, Germany). For hydrogen and oxygen, our approach involved the measurement of both *δ*^2^H and *δ*^18^O values with the same analytical run (*i.e*., both H_2_ and CO gases were analyzed from the same pyrolysis). Samples and standards were weighed to 350 ±20 *μ*g in silver capsules and analysed using a IRMS system (Thermo Finnigan, Bremen, Germany) equipped with a Costech Zero-Blank autosampler. The helium carrier gas rate was set to 120 mL/min. We used a HTC 1.5 m 0.25 inch 5 Åmolecular sieve (80-100 mesh) GC column. The glassy carbon reactor was operated at a temperature of 1400°C, and the GC column temperature was set to 90 °C. After separation, the gases were introduced into a Delta V Plus isotope–ratio mass spectrometer via a ConFlo IV interface (Thermo Finnigan, Bremen, Germany). The eluted N_2_ was flushed to waste by withdrawing the CF capillary from the ConFlo interface. We used Environment Canada keratin reference standards CBS (Caribou hoof) and KHS (Kudu horn) to calibrate sample *δ*^2^H (-197 and -54.1 per mil, respectively) and *δ*^18^O values (+2.50 and +21.46 per mil, respectively [24]). This normalization with calibrated keratins also corrects for any hydrogen isotope measurement artefact caused by production of HCN [25] in the glassy carbon reactor as described by Soto *et al*., 2017 [26]. Based on replicate (n = 5) within-run measurements of keratin standards and from historical analyses of an in-house QA/QC reference (SPK keratin), sample measurement error was estimated at ±2 per mil for *δ*^2^H and ±0.4 per mil for *δ*^18^O. All H results are reported for nonexchangeable H and for both H and O in the standard delta notation, normalized on the Vienna Standard Mean Ocean Water—Standard Light Antarctic Precipitation (VSMOW-SLAP) scale.

All water samples were measured for their stable isotopic compositions by Off Axis Integrated Cavity Output Spectroscopy (OA-ICOS) using a Los Gatos Research DLT-100 laser spectrometer. We used two calibrated reference waters (INV1 *δ*^2^H = -217.7, *δ*^18^O = -28.5 and ROD3 *δ*^2^H = -3.9, *δ*^18^O = -1.0 per mil, respectively) to normalize raw delta values to the VSMOW–SLAP scale. To minimize memory effects, samples and reference waters were injected nine times and the last five measurements were averaged to obtain the final raw delta values. Precisions as determined by replicate analyses of samples and reference waters were ± 1 and 0.1 per mil for hydrogen and oxygen, respectively. We used the R programming language for all statistical calculations [27].

## Results and discussion

**Fig 2:**
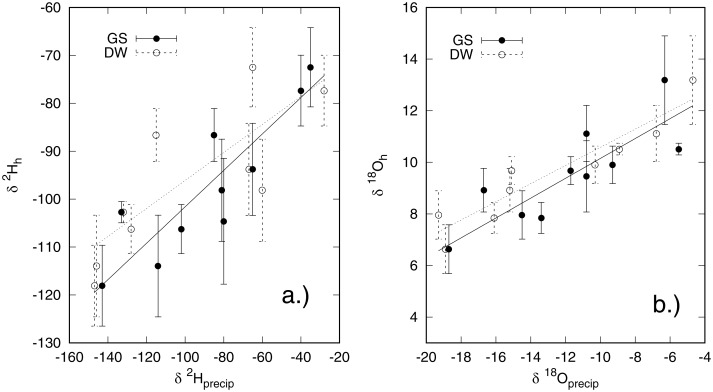
Relationship between cat (*F. catus*) hair a) *δ*^2^H values and b) *δ*^18^O values and those of growing season precipitation for North America (GS) and of drinking water (DW).

**Fig 3:**
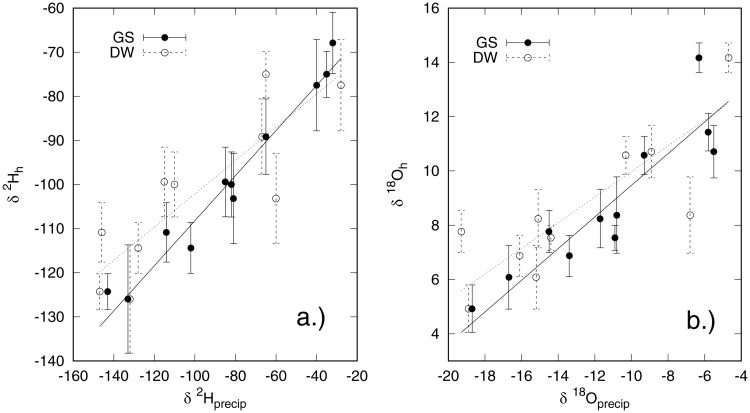
Relationship between dog (*C. lupus*) hair a) *δ*^2^H values and b) *δ*^18^O values and those of growing season precipitation for North America (GS) and of drinking water (DW).

We observe a strong positive relationship between *δ*^2^H and *δ*^18^O values of amount–weighted growing season average precipitation and those measured in hair for both cats and dogs (Table 1, Figs 2 and 3), with a better fit for dogs than cats. It is also evident that isotopic coupling between hydrogen and oxygen isotopes in precipitation and hair keratin is weaker for cats than for dogs (Table 1). A slightly weaker fit but similar isotopic coupling for both cats and dogs was measured between the *δ*^2^H and *δ*^18^O values of drinking water supplied by the shelter and those of the hair (Figs 2 and 3). This was unexpected, however on closer examination it was evident that most of the animals in the shelters had been there only a short time (<1 month), were all local animals, and many were feral, especially in northern Canada. Therefore, it is likely that these animals integrate water in their local environment in a similar way to what would occur in the wild. Moreover, there were a few instances where the drinking water isotopic compositions were markedly different than the local precipitation. An example of this is for Winnipeg, Canada, where the local tap water is sourced from the Shoal Lake reservoir and has a different stable isotopic composition than modeled local precipitation. For these reasons, we conclude that hair keratin hydrogen and oxygen isotopic compositions in this study are more closely correlated with growing season isotopic compositions of precipitation than of drinking water given to the shelter animals.

**Table 1:** RMA(orthogonal) linear regressions between hydrogen and oxygen isotopic compositions of growing season precipitation and hair keratin for cats and dogs. Subscripts h and p represent hair and precipitation, respectively.

Hydrogen	fit	r^2^	p
Cats	*δ*^2^H_*h*_ = 0.380*δ*^2^H_*p*_ − 63.6	0.769	< 0.001
Dogs	*δ*^2^H_*h*_ = 0.511*δ*^2^H_*p*_ − 57.1	0.953	<0.001
Oxygen			
Cats	*δ*^18^O_h_ = 0.385*δ*^18^O_*p*_ + 14.0	0.687	<0.001
Dogs	*δ*^18^O_*h*_ = 0.583*δ*^18^O_*p*_ + 14.3	0.814	<0.001

While the close relationship between the hydrogen and oxygen isotopic compositions of environmental water and hair keratin was expected for dogs, it contrasts sharply to previous work with cats. Pietsch *et al*. (2011) [20] did not measure any relationship between environmental water and hair keratin for cougars or bobcats, although a distinct relationship was found for the prey species, deer and cottontail rabbit. We have no explanation for this discrepancy, however we note that Pietsch *et al*. worked with museum specimens collected up to 142 years ago and which had been chemically preserved. Hair keratin is not affected by general tanning [28], however chemical treatments may vary, especially with historic samples. It is additionally possible that the hair may have been contaminated with other substances involved in the taxidermic process which resulted in erroneous isotopic compositions. Other rather remote possibilities include poorly constrained locations of museum specimens or that cougars and bobcats are sufficiently different from domestic cats that they are isotopically uncoupled from environmental water.

For hydrogen isotopes, our results are broadly consistent with the strong relationships found previously between H isotopes in environmental waters and those in human hair [29–32], bird feathers [33] and bat hair [34, 35]. For mammals, hydrogen in body water is obtained from drinking water, food water, and metabolized food. Because non-essential amino acids are synthesized *in vivo*, their hydrogen stable isotopic compositions reflect those of body water. Essential amino acids must be obtained from diet and therefore their *δ*^2^H values are dependent on those of food sources. As a result, animals that biosynthesize more amino acids will have a higher contribution of environmentally derived hydrogen in their tissues, including keratin. Cats are obligate carnivores and need higher amounts of specific essential amino acids in their diet (taurine, arginine, methionine, and cysteine) than do facultative carnivores, such as dogs, or omnivores [36]. This is most likely because these essential amino acids are abundant in prey and *in vivo* synthesis is not required because it is redundant [37]. Carbohydrates, such as starches and plant material, can be tolerated by dogs but not by cats [38].

For oxygen isotopes, a similar dependence on body water is expected except that carboxyl bound O is fully available during peptide hydrolysis and therefore oxygen can exchange with body water for both essential and nonessential amino acids. This suggests that oxygen isotope ratios in keratins may be insensitive to the extent of *in vivo* synthesis [32] but more affected by the relative contribution of body water and the isotopic fractionation between water and carbonyl oxygen during amino acid synthesis [39]. Close isotopic coupling of oxygen isotopes between ambient water and wing material has been reported for aquatic emergent insects such as dragonflies [40], but this coupling is not as evident in birds or terrestrial insects [19, 41].

Many mammals vary in their relative use of drinking water versus metabolic water as a contribution to the body water pool available for H and O isotopic exchange. For example, polar bears (*Ursus maritimus*) do not drink fresh water. Instead, they produce body water by consumption of a high fat diet, for which the catabolic products are only water and carbon dioxide [42, 43]. By contrast, the water needs of cats reflect both their evolution in arid environments and their development as strict carnivores. They can obtain most of their water requirements from consumption of prey, but with their high protein intake, they do need some water because the end products of protein catabolism (urea, ammonia, uric acid, and creatinine) must be excreted in urine. Dogs, unlike cats, do not concentrate their urine and thus their water intake needs are higher still, as are humans and other non water–conserving mammals [44]. Other metabolic differences specific to felids include a higher metabolism [45] or relatively poor heat loss response involving increased internal evaporation (*i.e*. panting) [46, 47], either of which may alter the hydrogen and oxygen stable isotopic compositions of body water, and thus hair.

In summary, the relative influence of H and O isotopes in environmental waters on hair keratin is expected to be affected by the extent of *in vivo* amino acid synthesis with more of an influence on hydrogen isotopes in cases of higher levels of synthesis. Because oxygen in body water is not only derived from drinking water and diet but also O_2_ in air through respiration [48], it is possible that the isotopic coupling of oxygen isotopes between hair and body water will be dependent on the relative use of these two pools [49], or like hydrogen, may be influenced by the relative role of hydration in overall nutrition. It is worth noting, however, that the transfer of hydrogen and oxygen from environmental water into animal tissues is a complex process and is still poorly understood. For instance, *δ*^2^H and *δ*^18^O values of local food sources also likely vary with those of precipitation so that it is difficult to accurately asses the relative contributions between food sources and drinking water outside the laboratory environment. All things considered, however, we would expect a weaker relationship between the *δ*^2^H and *δ*^18^O values of hair keratin and those of local precipitation for cats than for dogs, which is consistent with our data.

**Fig 4:**
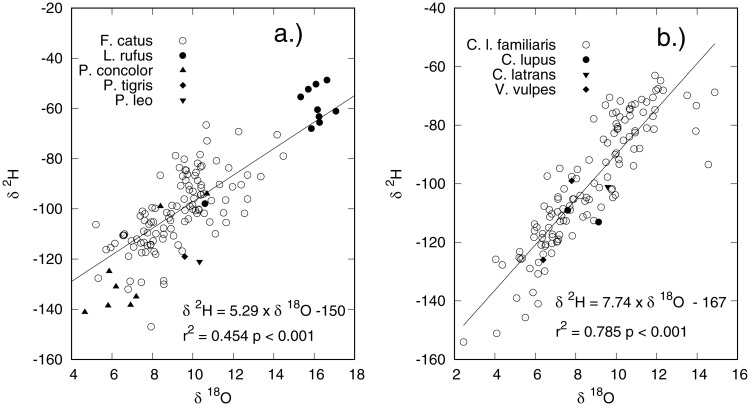
Relationship between a) cat (*F. catus*) and b) dog (*C. lupus familiaris*) hair *δ*^2^H and *δ*^18^O values. Also included are wild and captive bobcats, cougars, tigers, lions, coyotes, wolves, and foxes (*L. rufus, P. concolor, P. tigris, P. leo, C. latrans, C. lupus*, and *V. vulpes*).

The relationship between the *δ*^2^H and *δ*^18^O values of hair for cats and dogs are shown in Fig 4. For dogs, there is a good correlation (r^2^ = 0.785) between *δ*^2^H and *δ*^18^O values of hair with a regressed slope that is similar to the relationship for meteoric water. The *δ*^2^H and *δ*^18^O values of cats, on the other hand, display a weaker correlation (r^2^ = 0.454) and the slope of the regression line is shallower than we would expect for meteoric waters. This suggests that, on average, there is less relative transfer of hydrogen than oxygen isotopes from environmental waters into hair keratin. The reasons for this are unclear but most likely are the result of the dietary and metabolic differences inherent in the obligate carnivore physiology, as discussed previously.

Hydrogen and oxygen stable isotopic compositions from fur of both wild and captive large cats (tigers, cougars, bobcats, and lions) are broadly consistent with our data from domestic cats, although the sample size is small. Similarly, captive and wild canids (foxes, coyotes, and wolves) seem to follow the same trend as do domestic dogs (Fig 4). Moreover, our measurements of the relative isotopic contribution of oxygen from environmental waters to body water of 0.385 (Table 1) agrees well with those measured in bone phosphate from wild bobcats and cougars (0.41 and 0.38, respectively) [22]. While not definitive, these data suggest that coupling of hydrogen and oxygen from environmental waters into tissues of wild felids and canids are likely similar to our observed measurements with domestic cats and dogs.

## Conclusions

Our findings open up considerable new possibilities for determining origins or tracking migration of high level carnivores, such as felids and canids, using isotopic methods as has been demonstrated for birds, insects, and other taxa [17]. This is especially important because many terrestrial large mammals, particularly the carnivores, are currently under threat and isotopic methods for tracking and provenance have been investigated for only a few species, such as humans and bats [50].

## Supporting information

S1 File Excel streadsheet of *δ*^2^H and *δ*^18^O values of measured hair, water and predicted precipitation. (XLSX)
